# Community engagement and life satisfaction amongst older adults in Chengdu, China: Moderated mediation by social capital, gender and age

**DOI:** 10.1111/ajag.70073

**Published:** 2025-08-08

**Authors:** Chien‐Chung Huang, Xiaoxia Xie, Yulu Tu, Xiaoyao Jiang

**Affiliations:** ^1^ School of Social Work Rutgers University New Brunswick New Jersey USA; ^2^ Research Institute of Social Development Southwestern University of Finance & Economics Chengdu China; ^3^ Faculty of International Studies Southwestern University of Finance & Economics Chengdu China

**Keywords:** aged, healthy ageing, life satisfaction, social capital

## Abstract

**Objective:**

To explore the association between community engagement and life satisfaction amongst older adults, with a focus on the mediating effect of social capital and the moderating effects of gender and age.

**Methods:**

This study used structural equation modelling to analyse data from a sample of 1036 older adults in Chengdu, China, examining both the direct and indirect effects of community engagement on life satisfaction through social capital, and whether the relations moderated by gender and age. Model fit was evaluated using *χ* ^2^, Comparative Fit Index, Root Mean Square Error of Approximation and Standardised Root Mean Square Residual to ensure the validity of the proposed relationships.

**Results:**

The findings indicated a significant positive relationship between community engagement and social capital (*β* = .54) as well as between social capital and life satisfaction (*β* = .28). Social capital fully mediated the association between community engagement and life satisfaction. Moderation analysis revealed that the effects of community engagement and social capital on life satisfaction were stronger amongst older males (*β* = .57 and .32) than older females (*β* = .52 and .26).

**Conclusions:**

This study highlights the significant role of community engagement in enhancing social capital and life satisfaction amongst older adults in China, particularly for males and individuals aged 70 years or older. Findings indicate that policymakers should prioritise the development and implementation of community engagement programs specifically designed to support older men and individuals aged 70 years or older. It underscores the importance of culturally specific strategies for promoting active ageing in China.

**Policy impact**

Promoting community engagement and social capital can significantly enhance life satisfaction in older adults, particularly older men and those over 70 years. Policies should support tailored programs in community centres, focusing on social networking and engagement to build social capital and improve life satisfaction, addressing gender and age disparities in outcomes.

## 1 INTRODUCTION

China has an ever‐growing older adult population that accounts for roughly 19% of its entire population.^1^, ^2^ By 2050, the older adult population is expected to account for 30% of the population.^1^ In this large population, older adults are experiencing unprecedented longevity, often with higher rates of chronic geriatric conditions compared to those in other countries.^1^, ^3^ Traditionally, Chinese culture emphasises family‐based caregiving for older adults, with minimal reliance on external resources.^2^ However, due to rapid urbanisation, modernisation and migration patterns, families are increasingly dispersed across the country, leaving many older adults without immediate family support.^3^ As a result, reliance on community‐based services has grown significantly.^2^, ^3^ Community centres play a key role in promoting social engagement, health and recreation for older adults, but their availability and effectiveness vary across regions.^1^, ^4^ However, since community‐based supports like centres or adult day care are new in much of China, especially rural areas, opportunities for active ageing vary by location.^4^, ^5^ In urban areas, some centres offer structured programs, yet many lack targeted interventions to boost social capital and life satisfaction. In rural areas, such supports remain limited or underdeveloped. Expanding community‐based ageing services is essential to support healthy, independent ageing and reduce reliance on welfare and health‐care systems.^1^

Life satisfaction is a common measure of subjective fulfilment in old age, predicting health and overall well‐being.^6^, ^7^ Satisfied older adults are more likely to maintain a functional, independent lifestyle enriched by social activities and personal hobbies.^2^ However, factors like gender and age can moderate life satisfaction.^2^, ^7^, ^8^, ^9^, ^10^ Amongst older adults, women generally report higher levels of life satisfaction compared to men. Additionally, age is positively associated with life satisfaction, although this relationship tends to follow a non‐linear pattern.^2^, ^9^, ^10^

While many studies have explored the effects of socioeconomic factors on life satisfaction in older adults, few have examined the impact of community engagement and social capital specifically amongst older adults in China. Given the increasing demands on China's welfare system and the need for community support services, this study investigated how social capital mediates the link between community engagement and life satisfaction. These insights may inform policymakers, social workers and health‐care providers in designing programs that foster active ageing. Additionally, we examined whether gender and age moderated these relationships.^2^, ^11^

### 1.1 Community engagement

Community engagement involves various ways people participate in their community across different life areas, such as domestic, social and civic domains.^12^, ^13^ This can include types of activities like volunteering, participating in local organisations, attending cultural events, engaging in political or advocacy efforts, and providing informal support to neighbours or other community members. For older adults, it fosters connections with friends, family and broader social networks, enhancing life satisfaction.^14^, ^15^, ^16^ Research shows community engagement boosts social support and self‐efficacy while reducing loneliness and distress, all of which are linked to improved life satisfaction.^17^, ^18^, ^19^, ^20^

### 1.2 Social capital

Social capital refers to an individual's sense of belonging and access to resources within social networks and relationships.^2^, ^21^, ^22^ It includes trust, social networks, membership and community connections. For older adults, social capital is vital as it offers supportive networks, encourages involvement and fosters a sense of meaning.^2^ This, in turn, enhances life satisfaction.^23^, ^24^, ^25^ Studies show a positive link between social capital and life satisfaction.^14^, ^26^ The relationship between social capital and life satisfaction tends to be stronger amongst older men than their female counterparts.^27^

### 1.3 Conceptual model and hypotheses

The ecological model of ageing suggests that ageing is shaped by the interaction of biological, behavioural and environmental factors.^28^, ^29^ It highlights the role of social and environmental influences, such as community engagement and social capital, in enhancing older adults' well‐being.^30^, ^31^ This aligns with the active ageing perspective, which emphasises optimising opportunities for health and participation to improve quality of life.^16^ Community engagement helps older adults build relationships and increase social capital, thereby boosting well‐being and life satisfaction.^32^ In addition, social capital theory argues that resources available within social networks and relationships can enhance development and life satisfaction of individuals.^21^, ^22^, ^33^

**FIGURE 1:**
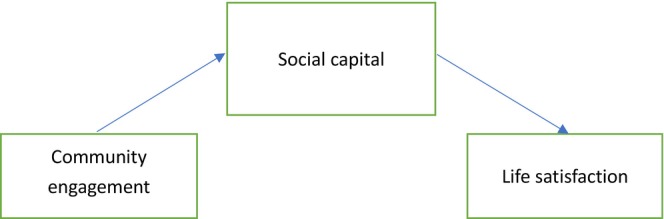
Conceptual model of community engagement, social support and loneliness.

This study was grounded in the ecological model, social capital theory and the active ageing perspective to (1) explore the impact of community engagement on life satisfaction; (2) investigate the mediating role of social capital in this relationship; and (3) examine whether gender and age moderate these effects. Given that social roles, opportunities for engagement and access to social capital often differ by gender and age, these factors may shape the extent to which community engagement influences life satisfaction.^2^, ^11^ The theoretical model is depicted in Figure 1. The study hypothesised: (1) Community engagement is positively linked to social capital; (2) Social capital is positively related to life satisfaction; (3) Social capital mediates the effect of community engagement on life satisfaction; and (4) Demographic factors (gender and age) moderate these relationships.

While prior research has shown the link between community engagement and life satisfaction through social capital, the mediating role of social capital and moderating effects of age and gender remain underexplored in older adults in China. This study aimed to address these gaps and offer insights for policies aimed at improving the well‐being of China's ageing population.

## 2 METHODS

This study employed a convenience sampling approach due to its feasibility in reaching a diverse sample of older adults engaged in community activities. Based on statistical data provided by the city government, five communities with a high proportion of retired older adults were selected for participant recruitment. The inclusion criteria for the sampling process required individuals to meet the following conditions: (1) residency in one of the five selected communities, (2) age 50 years or older and (3) retired from employment at the time of the survey. Individuals who were actively employed at the time of data collection were excluded from the study. Each selected community comprised approximately 250 retirees. With the assistance of local social workers and street‐level committees, surveys were distributed to retired older adults at local senior centres. From April 27 to June 27, 2022, we distributed 1167 questionnaires to older adults based in Chengdu, China. Of the 1167 surveys distributed, 1085 completed surveys were returned. However, 49 surveys were missing key data variables and were subsequently removed from the study. Our final sample size was 1036. The sample size exceeds the minimum required for detecting medium effect sizes (Cohen's *d* = .5) in regression analyses with 80% power at a .05 significance level, even when accounting for multiple predictors (*k* = 5–10), requiring around 60–100 participants. This ensured robust statistical power for detecting meaningful associations while accommodating subgroup analyses. Participants were informed that the survey was voluntary, with the option to withdraw at any time. Each received 3 RMB (0.5 USD) as compensation. The research protocol was approved by the Review Committee of the Research Institute of Social Development, Southwestern University of Finance and Economics (Protocol #2022002), and informed consent was obtained before starting the survey.

### 2.1 Measures

#### 2.1.1 Life satisfaction

Life satisfaction was measured using the 20‐item Life Satisfaction Index A (LSIA) by Neugarten et al. (1961), known for strong reliability and validity.^10^, ^34^ Participants rated statements on a 3‐point Likert scale (‘agree’ = 2, ‘uncertain’ = 1, ‘disagree’ = 0). Negatively worded items were reverse‐coded, with total scores ranging from 0 to 40. The Cronbach's alpha for the scale was .82.

#### 2.1.2 Community engagement

Community engagement was measured using eight items from a reliable and valid scale for Chinese populations.^35^ Participants reported the frequency of their interactions with neighbours, community organisations and governance committees on a scale from 1 (‘never’) to 4 (‘frequently’). Scores were averaged to create a community engagement score, with a Cronbach's alpha of .86.

#### 2.1.3 Social capital

Social capital was measured using the 42‐item Personal Social Capital Scale (PSCS),^36^ known for good reliability and validity.^37^ The PSCS includes 10 subconstructs, divided into bonding and bridging social capital (five each). Participants rated items on a 5‐point Likert scale (1 = ‘none’, 5 = ‘all’). Subconstruct scores were averaged to calculate bonding, bridging and overall social capital, with total scores ranging from 10 to 50. Cronbach's alpha values were .87 for bonding, .90 for bridging and .91 overall.

### 2.2 Analytical approach

We analysed the data using STATA version 16.0. First, descriptive statistics and Pearson's correlation were used to examine sample characteristics and variable relationships. We then applied structural equation modelling (SEM) with maximum likelihood estimation to assess the direct and indirect effects of community engagement on life satisfaction via social capital. Model fit was evaluated using *χ* ^2^, Comparative Fit Index (CFI) (>.95), Root Mean Square Error of Approximation (RMSEA) (<.08) and Standardised Root Mean Square Residual (SRMR) (<.08). We also tested the moderation effects of gender and age within the SEM. Additional regression analyses, controlling for relevant covariates, including gender, age, education, marital status and number of children, produced consistent findings. The SEM analysis was selected as the preferred analytical approach due to its capacity to simultaneously examine both direct and indirect effects of the mediating variable. In the social capital regression model, the community engagement coefficient was .5438 without covariates and slightly decreased to .5413 with covariates. Full results were available upon request.

## 3 RESULTS

The average age of the participants was 66 years (SD = 9), and the majority of the participants were between the ages of 60 and 69 (43%), followed by 70 years or older (32%), and then between 50 and 59 (26%) years old. About 56% of the participants identified as female. About 23% of the participants had below high school education, while 34% of them had a high school education. The percentages of the participants with vocational and college education degrees were 25% and 18%, respectively. The majority of the participants were married (86%), while the percentages of divorced and widowed participants were 6% and 8%, respectively. The average number of children was 1.4 (SD = .7). The participants demonstrated moderate levels of community engagement (M = 2.3, SD = .6, range: 1–4), life satisfaction (M = 25.1, SD = 6.5, range: 10–50) and social capital (M = 24.8, SD = 5.8, range: 0–40). The descriptive statistics of the participants were generally comparable to those of the broader population of retired and older adults in Chengdu, with the exception of socioeconomic characteristics, which were relatively more advantaged. For instance, 16% of older adults held a college degree in Chengdu's general population.^38^

The correlations for the key variables are presented in Table 1. The correlation analysis results supported our hypotheses. Community engagement was positively correlated with social capital (*r* = .54, *p* < .001) and life satisfaction (*r* = .17, *p* < .001). Similarly, social capital had a positive correlation with life satisfaction (*r* = .28, *p* < .01).

**TABLE 1:** Descriptive statistics and correlations of key variables.

	Mean (SD)	1	2	3
1. Community engagement [1–4]	2.3 (.6)			
2. Social capital [10–50]	24.8 (5.8)	.54***		
3. Life satisfaction [0–40]	25.1 (6.5)	.17***	.28**	

*Note*: *n* = 1036. Numbers in parentheses show ranges of the variables. **p < .01, ***p < .001.

**FIGURE 2:**
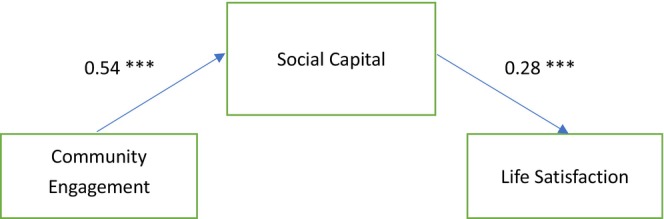
Standardised estimates of community engagement, social capital and life satisfaction. ***p < .001.

The standardised estimates for the SEM model are shown in Figure 2. The model fit indices indicated a good fit: *χ* ^2^ (1) = .62, *p* > .05; CFI = 1.00; RMSEA = .00; SRMR = .01. Community engagement was found to have a positive and significant effect on social capital (*β* = .54, *p* < .001), supporting Hypothesis 1. Consistent with Hypothesis 2, social capital directly and positively influenced life satisfaction (*β* = .28, *p* < .001). The SEM analysis revealed that social capital fully mediated the relationship between community engagement and life satisfaction. Additionally, an indirect effect of community engagement on life satisfaction through social capital was observed (*β* = .15, *p* < .001), supporting Hypothesis 3.

Table 2 lists the results of the moderation analysis. Likelihood‐ratio tests showed that the effects of community engagement and social capital on life satisfaction were significantly moderated by both gender and age. Specifically, the impact of community engagement on social capital was stronger for older male participants compared to female participants (.57 vs. .52), and for individuals aged 70 years or older compared to those aged 60–69 years (.62 vs. .49). Similarly, the influence of social capital was greater for older male participants compared to females (.32 vs. .26), and for those aged 70 years or older compared to participants aged 60–69 years (.40 vs. .29) or younger than 59 (.12). Furthermore, the indirect effect of community engagement on life satisfaction via social capital was more pronounced for adults aged 70 years or older (.25) and for male participants (.18).

**TABLE 2:** Direct and indirect effects of community engagement and social support on life satisfaction.

Independent variable	Dependent variable	Direct effect	Indirect effect
All sample			
Community engagement	Social capital	.54***	
Community engagement	Life satisfaction		.15***
Social capital	Life satisfaction	.28***	
Gender			
Male			
Community engagement	Social capital	.57***	
Community engagement	Life satisfaction		.18***
Social capital	Life satisfaction	.32***	
Female			
Community engagement	Social capital	.52***	
Community engagement	Life satisfaction		.14***
Social capital	Life satisfaction	.26***	
Likelihood‐ratio test		13.5*	
Age			
50–59 years			
Community engagement	Social capital	.52***	
Community engagement	Life satisfaction		.06***
Social capital	Life satisfaction	.12*	
60–69 years			
Community engagement	Social capital	.49***	
Community engagement	Life satisfaction		.14***
Social capital	Life satisfaction	.29***	
≥70 years			
Community engagement	Social capital	.62***	
Community engagement	Life satisfaction		.25***
Social capital	Life satisfaction	.40***	
Likelihood‐ratio test		25.6**	

*Note*: *n* = 1067. *p < .05, **p < .01, ***p < .001.

## 4 DISCUSSION

The results of the SEM analysis indicate that both community engagement and social capital are crucial factors for life satisfaction in older adults. Community engagement demonstrated a substantial impact on enhancing social capital, and social capital had a moderate effect on improving life satisfaction, aligning with previous studies.^2^, ^30^, ^31^ These findings align with the ecological model of ageing, which emphasises the dynamic interaction between individuals and their social and physical environments. The results suggest that older adults' engagement in community activities fosters social capital, which in turn enhances life satisfaction—illustrating how environmental and behavioural factors interact to influence ageing outcomes.^29^ Furthermore, the findings support social capital theory, as they demonstrate how increased social capital—characterised by trust, reciprocity and social networks—can facilitate higher levels of community engagement and well‐being amongst older adults. This underscores the role of social relationships in promoting active participation and life satisfaction in later life.^21^ Lastly, the results contribute to the active ageing perspective, which highlights the importance of continued participation in social, economic and cultural activities for maintaining well‐being. By showing that community engagement and social capital are positively associated with life satisfaction, our findings reinforce the notion that social participation is a key component of successful ageing.^16^, ^28^

These findings have practical implications. First, community centres should integrate services and programs that increase community engagement. Programs that encourage social networking and trust building improve social capital, which consequently can help older adults improve their life satisfaction.^2^, ^39^ As the ageing population in China continues to rapidly increase, it is crucial to expand community‐based interventions that enhance connection and well‐being amongst older adults.^40^, ^41^, ^42^, ^43^

Second, active ageing programs should consider improving social capital amongst older adults. Well‐designed social capital focused interventions can lead to improved physical and mental health amongst older adults.^44^, ^45^ For example, research indicates that the use of technologies, such as the internet and smartphones can enhance social capital amongst older adults.^46^, ^47^, ^48^ However, given that technology may not be accessible or appropriate for all individuals, programs integrating technological components should be implemented thoughtfully and adapted to align with the specific needs and capacities of older adult populations.

Third, the results of the moderation analysis suggest that the effects of community engagement and social capital had stronger effects on life satisfaction for older adults aged 70 years or older. Thus, government agencies can target this age demographic specifically to have the most effective impact. Additionally, the findings indicate that community engagement and social capital had larger effects on life satisfaction for older males, possibly due to men's greater risk of social isolation following retirement and their reliance on limited social networks.^27^, ^49^, ^50^, ^51^ To address these gender differences, programs could consider promoting structured activities for men and building on existing social networks for women. A gender‐sensitive approach may better enhance social capital and well‐being for all older adults.

This study had several limitations. First, the cross‐sectional design restricted our ability to determine causal relationships between community engagement and social capital. Future research could benefit from using a longitudinal approach to better explore the causal dynamics between these variables. Second, convenience sampling may introduce selection bias and limit the generalisability of findings. Future research should consider probability sampling to enhance the representativeness of the sample. Third, the participants in this study were recruited from local community centres. This recruitment approach may introduce selection bias, potentially overrepresenting older adults who are more socially active. Thus, the findings may not fully capture the experiences of less socially active individuals. Future research should employ more representative sampling methods to enhance the external validity of the results. Fourth, while this study employed SEM to examine the relationships amongst key variables, potential confounding factors that were not accounted for in the analysis may influence the findings. Unmeasured variables, such as personality traits and health conditions might contribute to variations in social capital, community engagement and life satisfaction. Future research should incorporate additional control variables to further assess the robustness of the estimated relationships. Additionally, the data relied on self‐reported responses from older adults residing in Chengdu, China. While self‐reported data are common in research, it may be subject to biases that can influence the findings. Finally, the sample for this study was limited to Chengdu, China, one of the country's largest cities, with a population exceeding 20 million in 2021. The generalisability of the findings to cities of different sizes and rural communities remains uncertain. Future research could explore the impact of geographic differences (e.g. rural areas, small and midsize cities) on community engagement, social capital and life satisfaction.

## 5 CONCLUSIONS

This study examined the relationship between community engagement, social capital and life satisfaction amongst older adults in Chengdu, China, with attention to age and gender differences. By focusing on a non‐Western context, it contributes to the broader understanding of social engagement and well‐being in later life. The findings underscore the importance of community engagement in enhancing life satisfaction through social capital, particularly for older males and adults aged 70 years or older. These insights highlight the need for targeted, community‐based interventions that address the diverse needs of ageing populations and promote social connectedness as a pathway to well‐being.

## FUNDING INFORMATION

This research received no external funding.

## CONFLICT OF INTEREST STATEMENT

No conflicts of interest declared.

## Data Availability

The datasets generated during and/or analysed during the current study are available from the corresponding author on reasonable request.
